# Help-seeking behavior of inmates in norwegian prisons

**DOI:** 10.1192/j.eurpsy.2021.1254

**Published:** 2021-08-13

**Authors:** L. Solbakken, S. Bergvik, R. Wynn

**Affiliations:** 1 Department Of Clinical Medicine, UiT The Arctic University of Norway, Tromsø, Norway; 2 Department Of Psychology, UiT The Arctic University of Norway, Tromsø, Norway

**Keywords:** help-seeking, health promotion, health literacy, inmates

## Abstract

**Introduction:**

While prison inmates have an increased risk of mental illness, psychiatric services are often less accessible and insufficient for this group. A low level of awareness or a fear of becoming stigmatised could also influence the help-seeking behaviour of some inmates.

**Objectives:**

To study the knowledge and beliefs regarding mental health and mental illness as well as the help-seeking behaviour of inmates in Norwegian prisons.

**Methods:**

We describe a study of help-seeking behaviour and mental health literacy of prisoners. This is a qualitiative study involving in-depth interviews with inmates in prisons in North Norway.

**Results:**

Recruitment and data collection is ongoing. Central topics in the interviews are inmates’ associations regarding positive mental health and how they can enhance their own well-being while in prison, and how other external factors can contribute to increased well-being. Furthermore, the inmates are asked about their attitudes, beliefs, and knowledge regarding mental illness, and what they think might be factors that can contribute to the development of mental illness. Moreover, we cover topics such as the inmates’ beliefs regarding the treatment of mental illness, strategies for handling such health problems, and sources of information regarding mental health and mental illness.

**Conclusions:**

The study will increase knowledge about how prisoners think about mental health and mental illness and the help-seeking behaviours of prison inmates. In a next step, this understanding can be utilized in improving information about well-being, mental illness, and psychiatric services to prisoners.

